# The EADGENE and SABRE post-analyses workshop

**DOI:** 10.1186/1753-6561-3-S4-I1

**Published:** 2009-07-16

**Authors:** Florence Jaffrezic, Jakob Hedegaard, Magali SanCristobal, Christophe Klopp, Dirk-Jan de Koning

**Affiliations:** 1 INRA AgroParisTech, Animal Genetics and Integrative Biology, Populations Statistics Genomes, 78350 Jouy-en-Josas, France; 2 Aarhus University, Faculty of Agricultural Sciences, Department of Genetics and Biotechnology, P.O. Box 50 DK-8830 Tjele, Denmark; 3 INRA, UMR444 Laboratoire de Génétique Cellulaire, F-31326 Castanet-Tolosan, France; 4 Sigenae UR875 Biométrie et Intelligence Artificielle, INRA, BP 52627, 31326 Castanet-Tolosan Cedex, France; 5 Roslin Institute and R(D)SVS, University of Edinburgh, Roslin, EH25 9PS, UK

## Background

Analysis of genome-wide gene expression using DNA microarrays has become pervasive in almost all areas of biology. The area of biology addressed by this workshop is gene expression studies in livestock looking at transcriptomic differences between treatments as well as genotypes and combinations of these. Two years ago, we organized a workshop to discuss the best approaches to analyze two-colour DNA microarray data in our area of research and the outcomes of that workshop have been published in 4 open access publications [1-4]. While there is currently a reasonable amount of consensus on the statistical analyses of a microarray experiment (i.e. getting a gene list), the subsequently analysis of the gene list is still an area of much confusion to many scientists.

During a three-day workshop in November 2008, we discussed five aspects of these so-called post analyses of microarray data: 1) re-annotation of the probe set on DNA microarrays, 2) pathway analyses to identify significantly affected biological processes from microarray results, 3) reverse engineering of regulatory networks from microarray results, 4) the integration of gene expression studies with QTL detection studies and 5) the prediction of phenotypic outcomes using gene expression results.

Prior to the workshop, we distributed two sets of data to the workshop participants. The first set of gene expression data deals with experimental challenge of chicken with two types of *Eimeria*. This experiment is described in some detail in one of the summary papers [5], while the actual data is available from ArrayExpress  under accession number E-MEXP-1972. The second experiment deals with the transcriptomic effects of adrenocorticotropic hormone (ACTH) treatment in two breeds of pigs. These gene expression results are available from Gene Expression Omnibus (GEO, , GSE8377 – DH06 Adrenal ACTH Sus scrofa).

## Observations

### Re-annotation of microarray probe set

Up-to-date annotation and target specificity is essential for functional analysis of microarray data. Three annotation pipelines were used to re-annotate 791 selected probes from the chicken microarray [6-8] and subsequently compared [9]. The main difference between annotation pipelines came from differences between the thresholds that were applied in order to link a probe to a certain type of annotation. It was recommended to have flexible thresholds in order to evaluate the effect of stringency and strike the right balance between reliability and coverage of the annotation.

### The application of pathway analyses

Several conceptually different analytical approaches, using both commercial and public available software, were applied by the participating groups to interpret the affected probes from the chicken experiment [10-15]. A total of twelve pathway related software tools were tested on the chicken data. The main focus of the approaches was to utilise the relation between probes/genes and their gene ontology and pathways to interpret the affected probes/genes. The lack of a well annotated chicken genome did limit the possibilities to fully explore the tools. The main results from these analyses showed that the biological interpretation is highly dependent on the statistical method used but that some common biological conclusions could be reached [5].

### Reverse engineering of regulatory networks

Graphical Gaussian models, as implemented in the R library GeneNet, were applied to 85 gene transcripts from the chicken experiment that were selected for their significance and lack of missing data. While a large number of significant relationships (edges) were found between these 85 genes, they could not be confirmed using pathway analyses because of limited annotation [16].

### Integration of microarrays with QTL results

Using the pig experiment, three groups evaluated different ways to link the gene expression results to QTL results: 1) co-location between differentially expressed genes and QTL results from the same experiments [17,18], 2) co-location between differentially expressed genes and QTL from the public domain, and 3) overlap between genes and QTL regions at the Pathway level: genes and QTL may not co-locate but differentially expressed genes hare enriched pathways with genes in the QTL region [19]. Because the pig has only a preliminary draft genome sequence, comparative mapping approaches were also used to compare QTL locations and differentially expressed genes. Because of very limited annotations, no meaningful pathway comparisons could be made.

### Phenotypic prediction from microarray data

The pig data has two treatments and two genotypes. In order to predict these grouping using the microarray data the authors used a Random Forest approach and also compared the classical Partial Least Squares regression (PLS) with a novel approach called sparse PLS [20]. All methods performed well on this data set. The sparse PLS outperformed the PLS in terms of prediction performance and improved the interpretability of the results. Both approaches are well adapted to transcriptomic data where the number of features is much greater than the number of individuals. Only a small number of genes (<20) was required to give perfect prediction of the four groups.

### Take home message

The central theme of the meeting was the lack of annotation. This was not in terms of bioinformatics tools to link sequences between species but a clear lack of knowledge regarding gene function. This was not specific for livestock species and considerable efforts are required before pathway based approaches will really come to fruition. In this context, there is a clear benefit for methods that do not require any level of annotation such as reverse engineering of networks and phenotypic prediction from microarray data. One challenging opportunity is to catalogue this level of experimental annotation (e.g. 'up-regulated after infection with *Eimeria'*) as an alternative means to derive functional links over time.

## Competing interests

The authors declare that they have no competing interests.
